# Should higher-income countries pay their citizens to move to foreign care homes?

**DOI:** 10.1136/medethics-2020-106380

**Published:** 2021-03-22

**Authors:** Bouke de Vries

**Affiliations:** 1 Historical, Philosophical and Religious Studies, Umea Universitet, Umeå, Sweden; 2 Biomedical Ethics and Law, KU Leuven, Leuven, Belgium

**Keywords:** aged, public policy, health workforce, end of life care, political philosophy

## Abstract

Faced with relatively old and ageing populations, a growing number of higher-income countries are struggling to provide affordable and decent care to their older citizens. This contribution proposes a new policy for dealing with this challenge. Under certain conditions, I argue that states should pay their citizens to move to foreign care homes in order to ease the pressure on domestic care institutions. This is the case if—but not necessarily only if—(1) a significant proportion of resident citizens do not currently have access to adequate aged and nursing care; (2) the care in the foreign care homes is not worse than the one that is available in domestic care homes; (3) sending states conduct regular checks to ascertain that the level of care abroad is not worse or delegate this task to reliable local monitoring bodies; (4) appropriate measures have been taken to ensure that this type of migration does not harm local residents; and (5) the public money spent on the payments is not better spent on other ways of easing the pressure on domestic care institutions. I end by defending the proposed payments against the objection that they create morally problematic inequalities by exerting greater pressure on members of lower socioeconomic classes to migrate than on their more affluent compatriots.

## Introduction

Many higher-income countries are struggling to provide adequate and affordable care to their older citizens as a result of population ageing.^1^ Apart from the fact that the citizens of these countries live longer than ever before, which comes with a reduction in cognitive and physical abilities and an increased susceptibility to dementia and other age-related diseases, there are progressively fewer younger adults around to look after older generations due to declining birth rates.^2^ In this article, my aim is to propose a novel measure for dealing with this challenge. I will argue that there are cases where states should *pay* their resident citizens to move to foreign care homes in order to ease the pressure on domestic care institutions. Such cases exist if, but not necessary only if, (1) a significant proportion of resident citizens do not currently have access to adequate aged and nursing care; (2) the care in the foreign care homes is not worse than the one that is available in domestic care homes; (3) sending states conduct regular checks to ascertain that the level of care abroad is not worse or delegate this task to reliable local monitoring bodies; (4) appropriate measures have been taken to ensure that this type of migration does not harm local residents; and (5) the public money spent on the payments is not better spent on other ways of easing the pressure on domestic care institutions. I end by defending the proposed payments against the objection that they create morally problematic inequalities by exerting greater pressure on members of lower socioeconomic classes to migrate than on their more affluent compatriots.

## Some preliminaries

Before discussing the pros and cons of paying resident citizens to live in foreign care homes, four preliminary comments are in order.

### Eligibility

The first is that, throughout this article, my focus is on cases where such payments are made to citizens who have made *personal decisions* to live in foreign care homes. This excludes cases where people have been sent to such care homes by others (eg, relatives) because they were no longer cognitively capable of deciding about a move abroad. The reason for focusing on this group is that paying expatriated individuals is especially controversial as it creates a risk that people will be sent abroad for simple financial gain, such as when an adult child sends her parent to a low-cost foreign care home in order to receive a larger inheritance later. (This, to be sure, does not necessarily mean that those who are expatriated should not be paid. One potential problem with not doing so is that these individuals will receive less financial support than their sedentary counterparts, which might create an objectionable inequality between citizens; another potential problem is that being denied these benefits might deprive poor expatriated citizens of the wherewithal to live a minimally decent life.)

### Types of payments

The second comment is that the proposed payments may take different forms. One involves paying citizens *once they have moved to a foreign care home*. This may be done by transferring money to them or to a legally authorised attorney directly. However, it might also be done by subsidising rental costs as well as by providing in-kind benefits that cover specific medical and health-related needs that the emigrants might have. I will call such payments ‘synchronous payments’.

The other form that the proposed payments may take is that citizens who do not currently need a care home place are paid *in advance for living in a foreign care home should they come to require residential care in the future*. The strictest version of this approach would make it illegal for recipients of these payments to live in any domestic care home so as to maximise the number of domestic care home places for citizens who did not agree to, and therefore did not receive, said payments. However, more moderate versions can be envisaged on which states simply withdraw the right to reside in publicly owned care homes but not the right to reside in privately owned ones.

The main advantage that advance payments have over synchronous payments is that they allow the recipients to spend the money during a time of their life where it might bring them greater benefits than when they are indigent, which may because they are able to use the money to partially fund a new house, send their children to a better school and go on long haul holidays. At the same time, advance payments raise certain problems that do not plague synchronous payments. The most important one is that citizens who have been paid for moving to a foreign care home ex ante might refuse to do so once their deteriorating health and abilities force them to live in a care home (rather than delving into this issue here, which would take us too far afield, I will just note that states could respond to it by requiring such undeserved benefits to be paid back or by refusing certain welfare benefits as compensation). For the purposes of this article, I shall remain non-committal on whether states with moral duties to pay their citizens to live in foreign care homes should offer only one of these types of payments—and, if so, which one—or rather both types.

### Size of the payments

The third comment is that in cases where states ought to be paying their citizens to live in foreign care homes, the amount that they should pay them will vary. Factors on which it depends include, but are not necessarily limited, the state’s level of wealth, the magnitude of the strain on its aged and nursing care systems, the amount of public money that is saved when citizens live in specific foreign care homes, the amount of public money that is necessary to convince different groups of citizens to live in foreign care homes, the amount of public money that is necessary to monitor the quantity and quality of the care abroad (about which more in the Monitoring foreign care homes section), and the amount of public money that is necessary to off-set any harm that this type of migration might cause to local inhabitants (about which more in the Protecting local inhabitants section).

### Repatriation options

The final comment is that I am not ruling out, but neither am I defending in this article, that citizens who are currently residing in foreign care homes and who are and/or have been paid for this should be (eventually) allowed to move to a privately owned domestic care home and possibly also to a publicly owned domestic care home as, for example, those suffering from persisting home sicknesses might wish to do.[1] I have parenthesised the word ‘eventually’ because in cases where citizens have received advance payments, domestic care home places—or at least ones in publicly owned care homes—might need to be refused to them for a certain period after they have moved abroad. Failing to do so may mean that some people will move to foreign care homes *without* the intention of staying there just so that they do not have to pay back the money that they have received for agreeing to live abroad. (Notice that the problem of such fraud does not arise with regard to synchronous payments, given that under this model, people are only paid once, and for as long as, they live in a foreign care home.)

## When states should pay citizens to move to foreign care homes

With these preliminaries out of the way, my aim in this section is to propose five conditions under which it is not just morally permissible but *morally required* for states to pay their citizens to live in foreign care homes before answering some criticisms of these payments within the next section.

### Inadequate aged and nursing care

The first condition is that a significant proportion of resident citizens do not currently have access to adequate aged and nursing care. This condition is satisfied within many higher-income countries on which I focus from hereon, given that most people who move to a foreign care home come from these countries (to see why, notice that while citizens of higher-income countries tend to save money by living in care homes within lower-income countries, the opposite does not hold). Using data from the USA, Canada, England, Germany, Norway and Sweden, a cross-national study from 2012 found that nurse staffing standards and staffing levels were lower than experts recommended in all but the two Scandinavian countries.^1^ More recent evidence of the inadequacy of the institutional care within some of these countries is offered by health economist Heinz Rothgang. In a 2020 report, he and his colleagues found that residents of German nursing homes currently receive a daily average of 99 min of care, whereas they are estimated to require a daily average of 141 min in order to enjoy a minimally decent living standard.^3^ One might also consider a recent British survey among 1544 care home workers that found that abuse of residents by overwhelmed care home staff is a pervasive phenomenon in English care homes,[2] one that was reported to occur in 91 out of 92 surveyed homes.^4^ When we take into account that all higher-income countries are ageing and will continue to do so in the coming years and decades,^5^ for example, the share of Germans aged 60 years and above is expected to rise from 27% in 2014 to 35 % in 2030 and to 38% in 2050,^6^ while the proportion of English people aged 65 years and over is projected to increase from 18.2% in 2018 to 20.7% in 2028^7^—it becomes clear that these problems are unlikely to disappear in the foreseeable future without (further) state measures.

### Living standards within foreign care homes

There are many philosophers who accept—as do I, although a defence of this claim is beyond this article’s scope—that states have moral duties to help ensure that their citizens can live minimally decent lives understood as ones in which people are able to satisfy all of their basic interests, including ones in adequate care. Suppose that they are right to believe in such duties. Even then, the fact that many higher-income countries are currently failing to make adequate aged and nursing care available (see the previous subsection) does not entail that they should be paying their citizens to move to foreign care homes in order to allay the pressure on domestic care institutions. For this to be the case, several additional conditions need to be satisfied.

One is that the care in the relevant foreign care homes is not worse—as determined by both its quantity and quality—than the one that is available in domestic care homes. To see the need for this condition, notice that by financially incentivising citizens to move to foreign care homes with living standards that are further below the adequacy threshold than the living standards of domestic care homes, states would be violating their duties to help ensure that their citizens can live minimally decent lives, given that those who end up moving to these care homes will find it more difficult to live a minimally decent life than if they had moved to a domestic care home. On this view, just as it would be unacceptable to pay citizens for waiving their right to, say, clean drinking water or to drinking water that is as close as possible to being clean, so it is unacceptable to pay them for waiving their right to live in a (publicly owned) domestic care home when such care homes offer them the best chances of living minimally decent lives in old age.

### Monitoring foreign care homes

Besides the need for the care in foreign care homes to be either better or simply not further below the adequacy threshold than the care in domestic care homes, would-be sending states need to have *good evidence that this condition is satisfied*. In order to obtain such evidence, they might either conduct their own checks in foreign care homes or delegate this task to any reliable monitoring bodies that might exist within the would-be receiving societies. For states to financially incentivise citizens to live in foreign care homes when there is no good evidence that the care abroad is of a sufficiently high calibre would be unacceptable as it would involve them taking a gamble with their citizens’ lives. This is so especially when the would-be receiving countries have little, if any, formal training requirements for care home staff, as well as when they are located far away[3] since this makes it comparatively difficult and costly for people to check in person how their relatives abroad are faring.

### Protecting local inhabitants

Still another condition that needs to be satisfied is that host populations are not harmed by the arrival of foreign care home residents. One way in which such harms might be caused is that the latter take up scarce places in care homes that would otherwise be available to locals. Even if locals are not being discriminated against in terms of admission policies, care home places might become prohibitively expensive for many when prices are driven up by the ability of immigrants to afford higher rental costs. Another way in which host populations might be harmed by this type of migration is that it engenders, or simply contributes to, domestic brain and care drains whereby local medical specialists and care workers start offering their services to comparatively wealthy foreign clients as opposed to local clients,^8^ which is a phenomenon that has been witnessed within the related context of medical tourism.^9^

While these are serious problems, it seems to me that they can be avoided by sending countries.[4] In order to avoid reducing the number of care home places for locals, they might, for instance, subsidise the construction of affordable care homes for this group and/or build care homes within the receiving countries that partly if not wholly accommodate their own citizens. And in order to prevent local brain and care drains, they could, inter alia, subsize the education of aspiring local medical experts and care workers on the condition that these individuals commit to working in their country’s public health and aged care sectors for a certain period after they finish their education.

### No better alternatives

This brings us to a final condition that ought to be satisfied. According to this condition, any public money that is spent on paying citizens to live in foreign care homes is not better entirely spent on other policies for providing aged and nursing care. Whether this is the case depends on how the effectiveness and moral and financial costs of these payments compare to the effectiveness and moral and financial costs of the relevant alternatives. Although an entire book could be written about this topic, what I want to suggest in the available space is that, within many higher-income countries, even the four most promising alternatives are unlikely to render the proposed payments superfluous.

#### Offering higher salaries to domestic care workers

This is a relatively expensive measure within higher-income countries. To bring this out, notice that because of the high work pressure within their aged and nursing care sectors, which is reflected in high attrition rates and the difficulties that many of these countries are experiencing in recruiting care workers among their existing populations (eg, some studies estimate that there will be a shortage of half a million care workers in Germany by 2030),^10^ it appears that a lot of extra money would need to be offered in order to convince enough people within society to become care workers. However, if this is correct, then even if many higher-income countries ought to be raising the salaries of domestic care workers, trying to meet the needs of their older populations entirely through the recruitment of domestic care workers is likely to be unreasonably expensive (assuming that, at some point, investments in aged and nursing care become too high relative to other important goods in which states ought to invest money, such as military security, children’s education and the environment).

#### Increasing child support

This can be a useful insofar as it helps to raise the birth rate and, correspondingly, to reduce the ageing of the population. However, it is not a short-term or a medium-term solution, given that it takes approximately 16 years before a newborn will be able to take up full-time employment.

#### Recruiting foreign care workers

While this measure increases the number of care workers in society and without any delay, it is vulnerable to some important problems. Not only does it require the immigrant care workers to live separated from their family, friends and country,^11^ which is something that would not be necessary if the care recipients moved to the immigrants’ countries instead (as they would do under the proposed solution), but also integrating these individuals will impose certain costs on receiving societies.^12^

#### Subsidising purchases of robot caregivers

Robot care givers are becoming increasingly sophisticated and already play a big role in providing care to people within countries such as Japan.^13^ However, it is unlikely that their abilities will soon match those of human care givers, apart from the fact that they raise certain moral challenges such as that they might be hacked by those with nefarious intentions and that some individuals with severe dementia do not realise that they are not sentient.^14^

## Objections and some rejoinders

The previous section has identified five conditions that I believe jointly impose moral duties on states to pay their citizens to move to foreign care homes. These conditions obtain when (1) a significant proportion of resident citizens do not currently have access to adequate aged and nursing care; (2) the care in the foreign care homes is not worse than the one that is available in domestic care homes; (3) sending states conduct regular checks to ascertain that the level of care abroad is not worse or delegate this task to reliable local monitoring bodies; (4) appropriate measures have been taken to ensure that this type of migration does not harm local residents; and (5) the public money spent on the payments is not better spent on other ways of easing the pressure on domestic care institutions. As was mentioned, the conditional duty to pay citizens to live in foreign care homes derives from a more general duty that I assume states to have, namely, that of helping to ensure that their citizens can live minimally decent lives. (Whereas a defence of this general duty is beyond this article’s scope, it was noted in the previous section that it is widely accepted among contemporary political philosophers even if more libertarian-minded theorists reject it.) To see how such payments help to discharge this duty within countries with overburdened aged and nursing care systems, it should be observed that the more citizens live in foreign care homes, the less pressure there will be on domestic care institutions, which means that the quantity and quality of the aged and nursing care within these countries will improve.

My aim in this final section is to answer a possible objection to the proposed payments. This objection has an empirical premise and a normative premise. Its empirical premise states that the relevant payments exert greater pressure on members of lower socioeconomic classes to migrate than on their more affluent compatriots, whereas its normative premise states that such inequalities are morally indefensible.

One immediate response to this objection is that when a society has realised a just distribution of socioeconomic resources and employment opportunities, it is not obvious that the fact that less resourceful individuals might face greater pressure to migrate than their more resourceful counterparts is actually problematic. To illustrate this, suppose that within a socioeconomically just society, there is a person who is willing to live within a lower-income country in old age to be able to afford his passion for globetrotting here and now. Now contrast this person with a conational who not only attaches great weight to spending the final years of her life within her country of residence and citizenship but also works long hours and sets aside money every year to be able to do so. In this case, it is all but clear that there is any injustice in the fact that the proposed payments put greater pressure on the globetrotter to migrate in old age than on his money-saving compatriot.

Some might retort that few, if any, contemporary higher-income countries have realised full socioeconomic justice and add that, under such non-ideal conditions, for the proposed payments to put greater pressure on members of lower socioeconomic classes to migrate than on members of higher socioeconomic classes is problematic. The reason for this, they may say, is that many of these individuals will be worse off in terms of their possessed wealth than they would be within a fully just society, which means that any disproportional pressure on them to migrate will be (partially) the product of unjust norms.

While this objection has some force, it does not count decisively against the proposed payments in any obvious sense. There are several reasons for this. First, even if these payments exert greater pressure on members of lower socioeconomic classes to migrate than on members of higher socioeconomic classes, the former are not being forced by their state to accept them. Second, such unequal pressure would be caused by the fact that members of lower socioeconomic classes not only have something to gain from the relevant payments but also have more to gain than members of higher socioeconomic classes, assuming diminishing marginal utility. However, if this is correct, then while any unequal pressure to migrate might be regrettable, it is far from clear that the right response is to deny a good to members of lower socioeconomic classes that is (particularly) valuable to them. This is so especially when we consider that even members of this group who would not accept the proposed payments, as a proportion of them are unlikely to given the depth of many people’s attachments to their country of residence, will derive important benefits from these payments. The reason for this is that when some of their conationals move to foreign care homes as a result of being paid to do so, domestic care workers will have more time to look after them as the caregiver:care recipient ratio will improve. Finally, there is a case to be made that, since some members of lower socioeconomic classes might move to foreign care homes *regardless* of whether they receive the proposed payments (reasons for which may include, but are not necessarily limited to, the fact that they can save on rental costs and/or enjoy a higher quality of care abroad), failing to let this group share in the benefits that their move brings to their sending society by paying them is unfair. In short, even when there is something untoward about the fact that paying citizens to live in foreign care homes is likely to put greater pressure on members of lower socioeconomic classes to migrate than on their more affluent compatriots, it is dubious whether this outweighs the strong reasons supporting such payments when the five conditions identified here are satisfied.

## Data Availability

There are no data in this work.

## Footnotes

There are real-life examples of such negative experiences; as Horn, Schweppe & Hollstein have found in a study among German and Swiss residents of Thai care homes, while some Germans and Swiss have very positive experiences in these care homes, others do not adjust well to their new life abroad.^15^

As Gröning and Lietzau discuss, comparable findings exist when it comes to the use of violence against care home residents in Germany.^16^

Think, for instance, of the distance between Germany and Thailand.

Notice that while receiving countries too can take measures to prevent these deleterious effects, my focus in this article is on sending countries.
